# Understanding Ureteropelvic Junction Obstruction From a Scientometric Perspective: How Is the Global Research Activity and Collaboration Going?

**DOI:** 10.1155/bmri/9969450

**Published:** 2025-11-03

**Authors:** Boshen Shu, Shufeng Zhang, Jian Gao, Lin Wang, Xiaohui Wang

**Affiliations:** ^1^ Department of Pediatric Surgery, Henan Provincial People’s Hospital, Zhengzhou, Henan Province, China, hnsrmyy.net

**Keywords:** collaboration, scientometrics, ureteropelvic junction obstruction, visualized study, VOSviewer

## Abstract

**Objective:**

Ureteropelvic junction obstruction (UPJO) has been identified as the most prevalent cause of prenatally diagnosed hydronephrosis, characterized by a reduction in urine flow from the renal pelvis into the ureter. Management of this condition poses a significant clinical challenge, given the difficulty in differentiating between mild and benign cases and those with the potential to progress to severe renal impairment. This study is aimed at clarifying the global research activity and collaborative trends in UPJO research by undertaking a scientometric analysis, with the objective of providing valuable insights into the state of UPJO research.

**Materials and Methods:**

Publications relevant to UPJO published between 1945 and 2024 were retrieved from the Web of Science Core Collection (WoSCC) database. The metrological software and graphing tools utilized included Bibliometrix 4.1.1, VOSviewer 1.6.20, GraphPad Prism 10, and Microsoft Excel 2020.

**Results:**

A total of 1447 publications between 1948 and 2024 were included. The United States contributed the most publications (*n* = 456, 31.5%), and the most productive journal is the *Journal of Urology* (*n* = 340, 23.5%). The latest keywords “classification”, “improvement”, and “assisted laparoscopic pyeloplasty” mainly appeared since 2020.

**Conclusions:**

In recent decades, UPJO research has undergone consistent advancement, encompassing a multitude of disciplines and innovative therapeutic strategies. The predominant research initiatives have been concentrated in a select number of high‐income countries. To facilitate further progress in this domain, there is a need to fortify international collaborations and promote translational research.

## 1. Introduction

Ureteropelvic junction obstruction (UPJO) is the most common cause of congenital hydronephrosis, affecting 1 in 1500 to 1 in 500 newborns, mostly males (2:1 ratio) [1, 2]. UPJO is typically a dilated pelvis without ureteral dilation, unlike obstructive megaureters or high‐grade vesicoureteral reflux, which present with dilated ureters. Severe UPJO requires surgery; open pyeloplasty (OP) was once the standard treatment for pediatric UPJO, with a reported success rate of over 90% [3]. However, minimally invasive surgical techniques have led to the development of laparoscopic pyeloplasty (LP), initially reported by Schuessler et al. in 1993 [4]. Since then, LP has emerged as the preferred procedure. The first robot‐assisted LP (RALP) was performed in 1999 by Yanke et al. [5]. After RALP was developed, single‐incision laparoscopic surgery and single‐incision‐plus‐one laparoscopic surgery were created for robot‐assisted single‐port‐plus‐one pyeloplasty and laparoscopic single‐port pyeloplasty, separately. Nonetheless, the management of UPJO has presented a conundrum for pediatric and adult urologists alike [2]. Surgical indications for symptomatic children are largely consistent, encompassing febrile urinary tract infections, progressive loss of function, and pain. However, the management of asymptomatic children remains debated, making it critical for clinicians and researchers to understand UPJO literature and scientific progress to inform future studies [1, 6]. Nevertheless, given the substantial quantity and heterogeneity of UPJO‐related publications, it is difficult for a single researcher to survey all the published items to discern the status, focal subjects, and emerging trends in UPJO research.

Scientometric study involves statistical analysis and assessment of published research, using visualized network analysis (e.g., bibliographic coupling and keyword co‐occurrence data) [7]. The primary domains of scientometric analysis encompass the assessment of research productivity and the measurement of citation impact within the scientific community [8]. It has been widely applied in diverse subjects to evaluate the impact of published studies or identify research trends [9, 10]. To date, a comprehensive study has not systematically analyzed the substantial body of literature related to UPJO research, thereby leaving the extent of the scientific output in this area ambiguous. Therefore, we aimed to systematically appraise the international UPJO research activity and collaboration via scientometric analysis, which may, in turn, contribute to the establishment of future cooperations and, consequently, the advancement of patient care.

## 2. Materials and Methods

### 2.1. Ethics Statement

Ethical approval was not required as no human or animal subjects were included in this analysis.

### 2.2. Data Source and Search Strategy

On January 10, 2025, we searched the Web of Science Core Collection (WoSCC) (Clarivate Analytics, Boston, Massachusetts, United States) for articles and citations related to UPJO published from its inception (January 1945) to December 2024. The “title” searching strategy was used to ensure the identification of relevant items [7, 11, 12]. This strategy aligns with established practices in relevant studies targeting focused clinical topics, where title‐based searches have been shown to balance sensitivity and specificity [7, 13]. The search terms were as follows: Title = “Ureteropelvic Junction Obstruction” OR “UP junction obstruction” OR “Obstruction of the ureteropelvic junction” OR “UPJO” OR “UPJ obstruction” AND publishing year = (1945.01.01–2024.12.31) AND Language = (English). As delineated in Figure 1a, the meticulous selection criteria and inclusive methodology of this study were exhibited in a systematic fashion. Titles, journals, authors, publication year, citation counts, organizations, countries, keywords, impact factor (IF), and *h*‐index were recorded and analyzed. The *h*‐index has been demonstrated as an established metric that incorporates a citation index and an institution’s or author’s overall scientific output. This enables the quantification of the importance, the impact, and the significance of an individual research contribution [14].

Figure 1(a) Flow diagram of the screening process concerning relevant publications on UPJO. (b) Publication and citation trends on UPJO.

**Figure:**
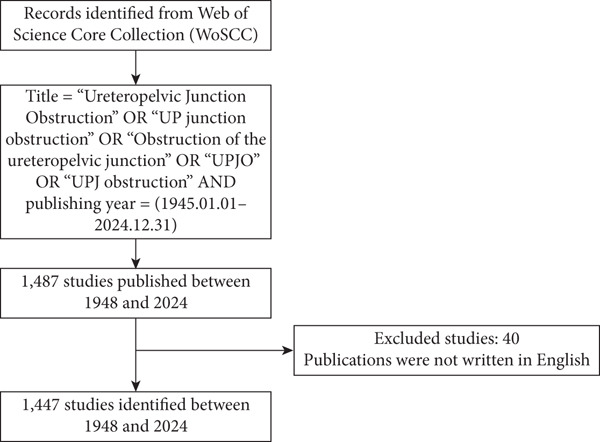
(a)

**Figure:**
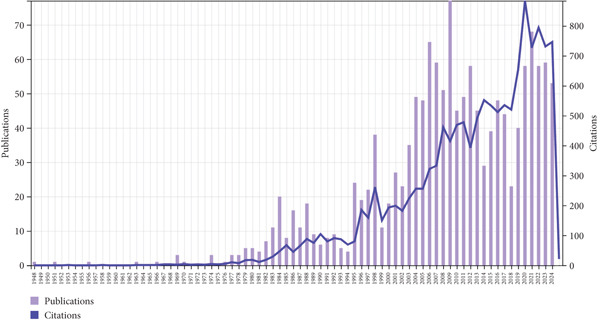
(b)

### 2.3. Scientometric Analysis

GraphPad Prism 10 (GraphPad, La Jolla, California, United States) and Microsoft Excel 2020 were used to analyze the scientometric characteristics for statistical analysis. VOSviewer 1.6.20 (Leiden University, Leiden, The Netherlands) and Bibliometrix 4.1.1 were used to execute the visualization analysis. The analysis evaluated collaboration networks among organizations, countries, and authors. “Hotspots” were identified using a co‐occurrence keyword assessment. Overlay visualization from VOSviewer and trend topics from Bibliometrix were applied to identify future research trends, and the line width and magnitude of dots in the network analysis map illustrated the total link intensity and frequency of articles. For VOSviewer, we used the “Leiden algorithm” as the clustering method. The threshold for keyword co‐occurrence analysis was set to a minimum of five occurrences, ensuring that only sufficiently frequent terms were included to avoid noise. For collaboration network analysis, the minimum number of documents per entity was set to 5, and total link strength was calculated using the default weighted association index. For Bibliometrix, the trend topic analysis was performed using the “growth rate” metric with a time window of 5 years, and the threshold for identifying significant trends was set to a minimum annual growth rate of 10%.

## 3. Results

### 3.1. Overview of Publications on UPJO

Based on our inclusion criteria, 1447 publications were identified from WoSCC, with a total of 13,682 citations (10,037 excluding self‐citations). The mean citation count per publication was 9.46, and the *h*‐index for all UPJO‐related articles was 51. The most‐cited article entitled “Predicting the clinical outcome of congenital unilateral ureteropelvic junction obstruction in newborn by urinary proteome analysis” was published in 2006 by Decramer et al. in *Nature Medicine* [15].

### 3.2. Year of Publication

The publication years of literature on UPJO ranged from 1948 to 2024, with the number of publications and citations per year both generally increasing (Figure 1b). The number of publications and citations per year reached the peak in the year 2009 (*n* = 77) and 2020 (*n* = 884), respectively.

### 3.3. Distribution of Journals

The *Journal of Urology* contributed the most publications on UPJO (IF = 6.4, *n* = 340, 23.5%), followed by the *Journal of Endourology* (IF = 2.9, *n* = 183, 12.6%) and *Urology* (IF = 2.1, *n* = 155, 10.7%) (Figure 2a). The top three journals published almost half of all included articles.

Figure 2(a) The top 10 contributing journals to UPJO research. (b) The distribution map of contributing countries on UPJO, deeper blue areas correspond to higher publication volumes for that country.

**Figure:**
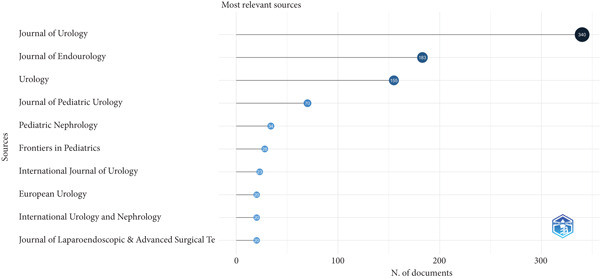
(a)

**Figure:**
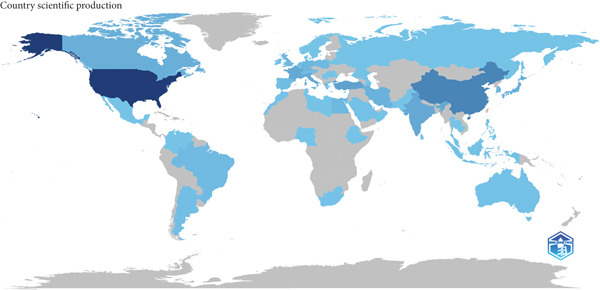
(b)

### 3.4. Analysis of Countries, Organizations, and Authors

A total of 66 countries were responsible for the selected 1447 studies pertaining to UPJO, the majority of which were in North America, Europe, and Asia. With respect to the countries of origin, the United States (*n* = 456) was the leading contributor, followed by China (*n* = 132) and India (*n* = 67) (Figure 2b). A global overview indicated 1085 organizations contributed to UPJO, with the top 10 institutions shown in Figure 3a. Quantitative analysis of authorship showed that Cadeddu JA contributed the most publications (*n* = 19), followed by Han SW (*n* = 16) and Smith AD (*n* = 15) (Figure 3b). In contrast, visual analysis of global collaboration revealed the United States as the leader, with the highest total link strength (Figure 4a). Cleveland Clinic and Xuesong L were identified as the organization and author with the strongest collaboration network (Figures 4b and 5a).

Figure 3(a) The top 10 contributing organizations to UPJO research. (b) The top 10 contributing authors to UPJO research.

**Figure:**
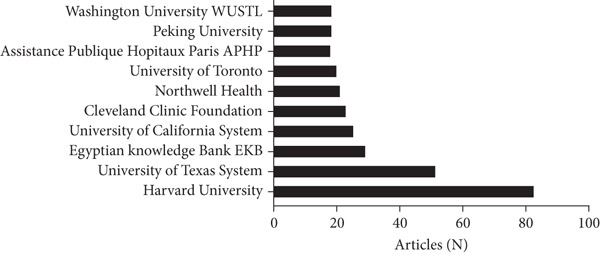
(a)

**Figure:**
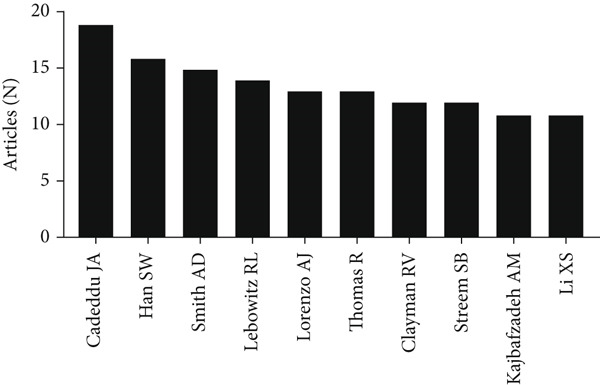
(b)

Figure 4(a) Global collaboration network on UPJO. (b) Bibliographic coupling analysis of institutions in UPJO research.

**Figure:**
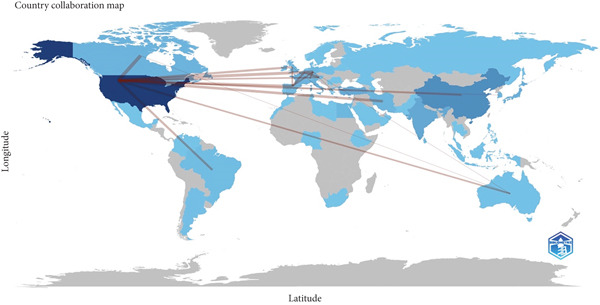
(a)

**Figure:**
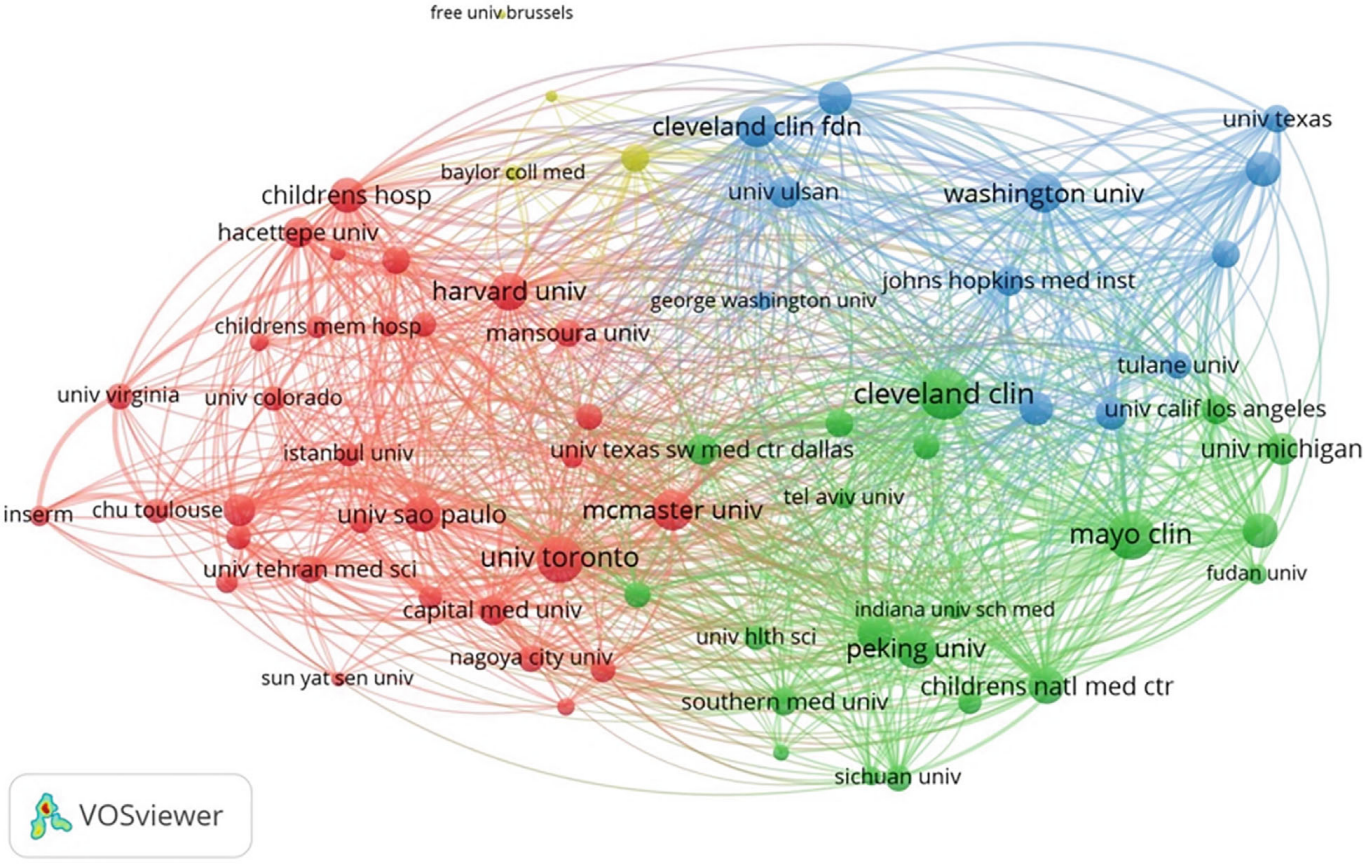
(b)

Figure 5(a) Bibliographic coupling analysis of authors in UPJO research. (b) Co‐occurrence analysis of keywords. (c) Keyword density map for co‐occurrence analysis.

**Figure:**
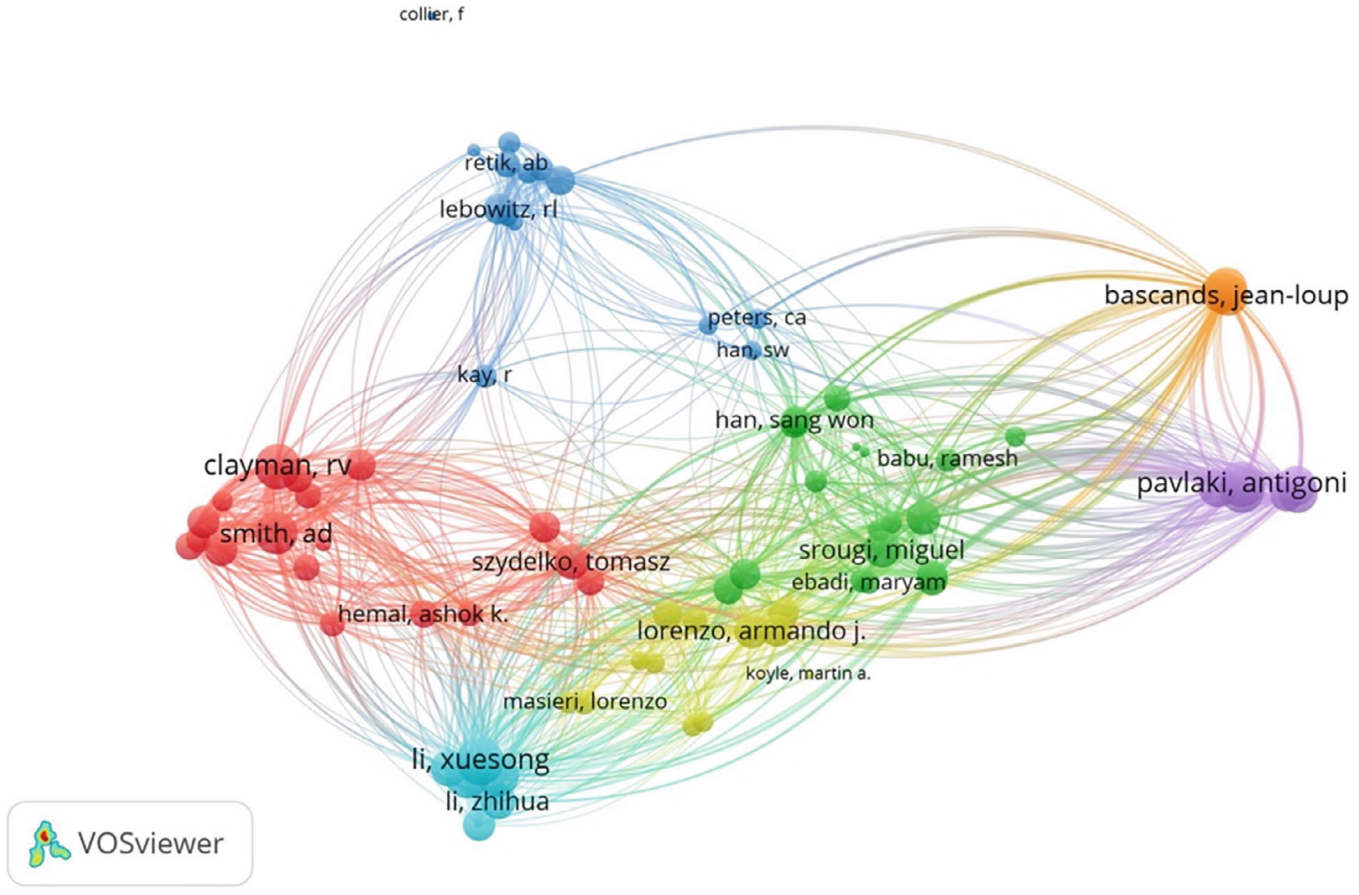
(a)

**Figure:**
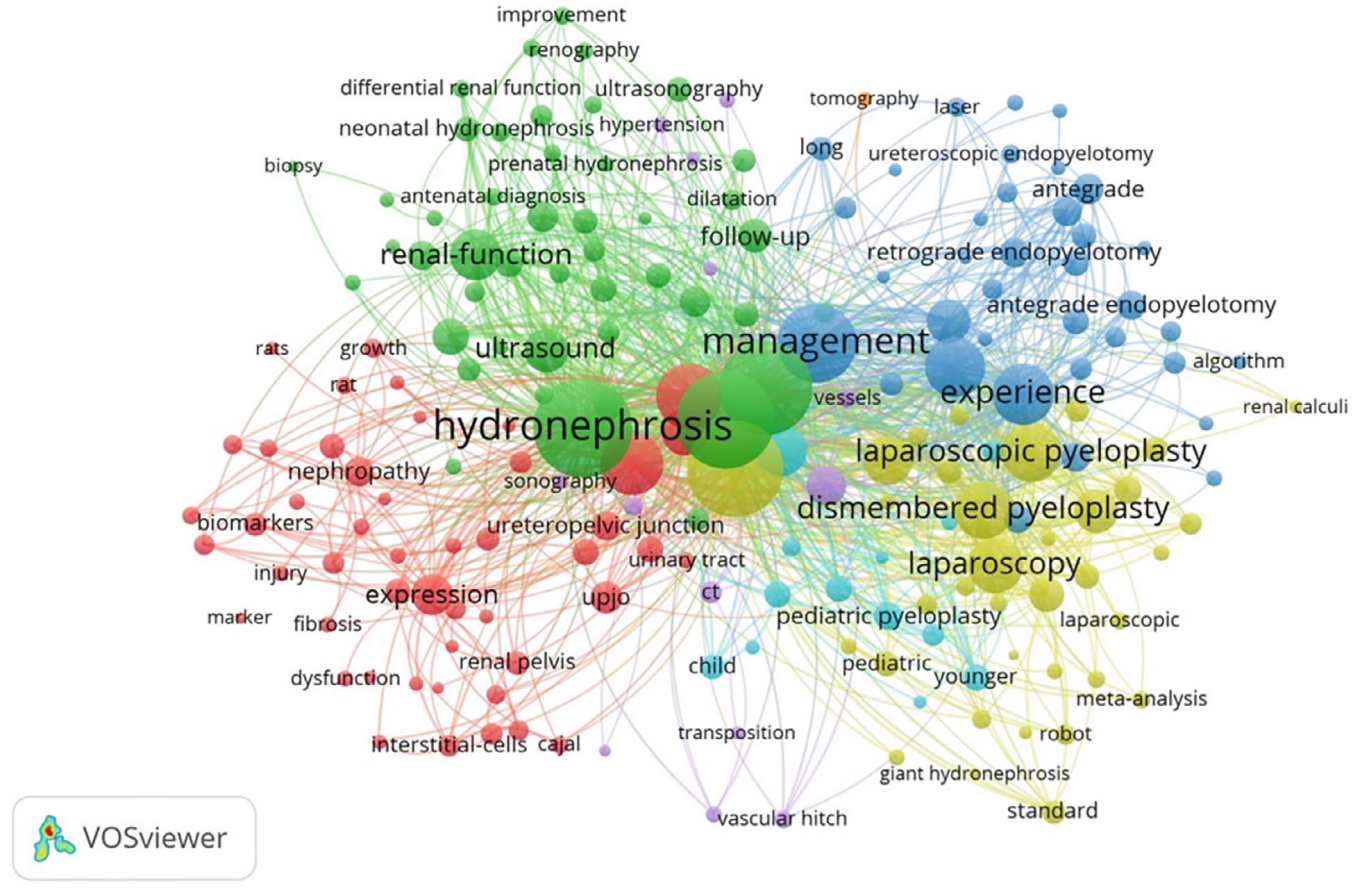
(b)

**Figure:**
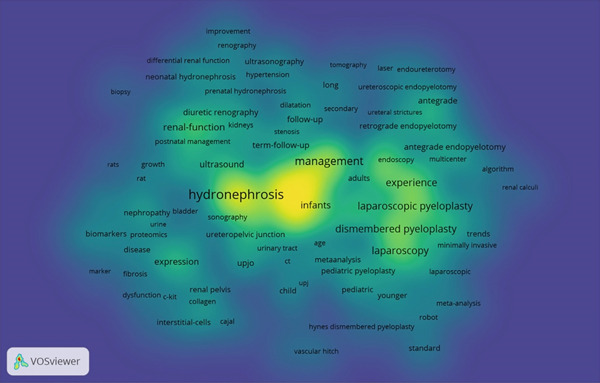
(c)

### 3.5. Maps of Co‐Occurrence Analysis

The minimum number of occurrences of a keyword was set at five of the 1717 keywords, and 192 met this threshold. The top three frequent keywords were “hydronephrosis”, “children”, and “pyeloplasty” with 253, 216, and 209 occurrences, respectively (Figure 5b,c). The most recent keywords, “classification”, “improvement”, and “assisted laparoscopic pyeloplasty”, emerged predominantly since the year 2020 (Figure 6a,b).

Figure 6(a) Time visualization on co‐occurrence analysis of keywords. (b) The analysis of research trend topics based on Bibliometrix in UPJO research.

**Figure:**
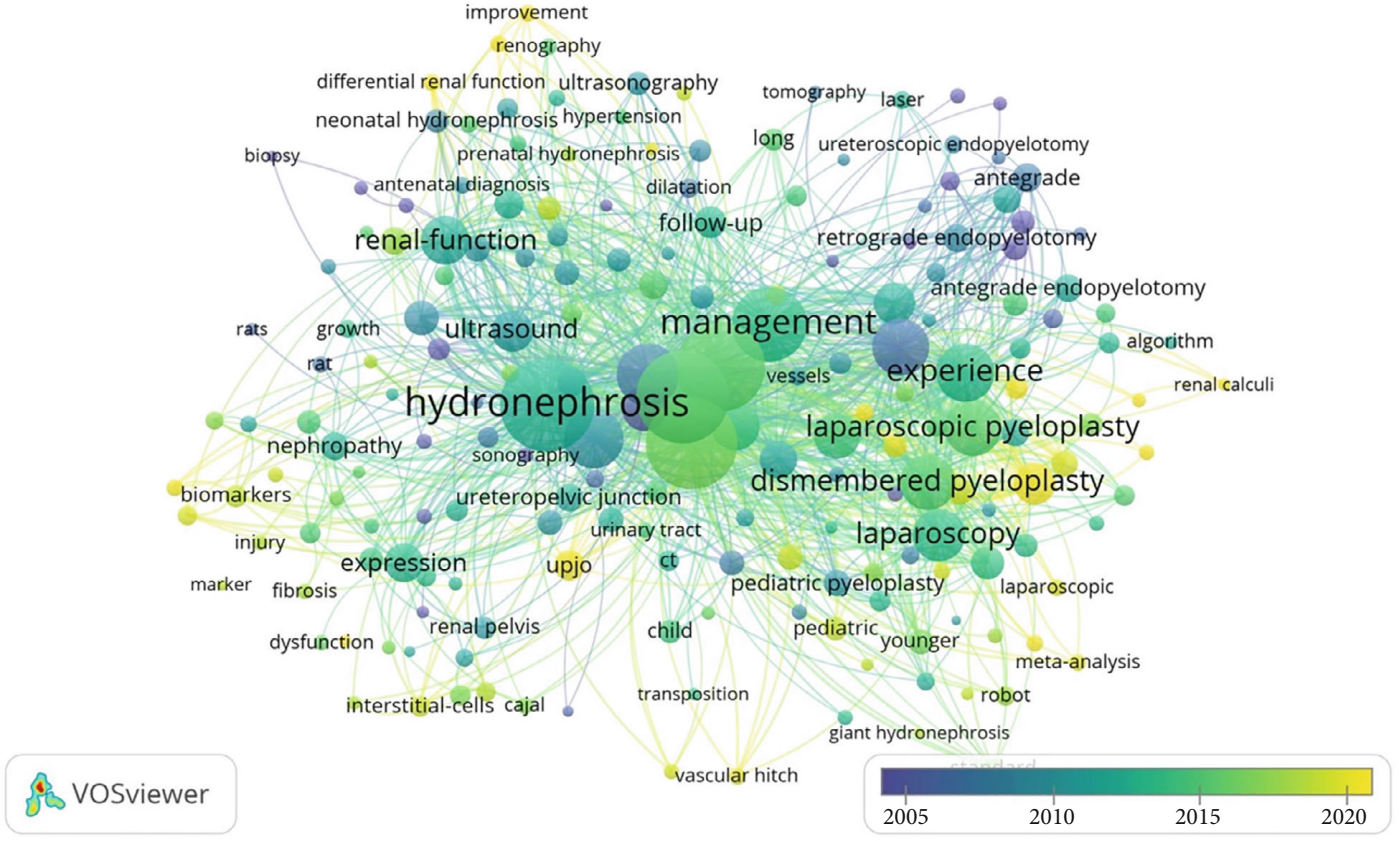
(a)

**Figure:**
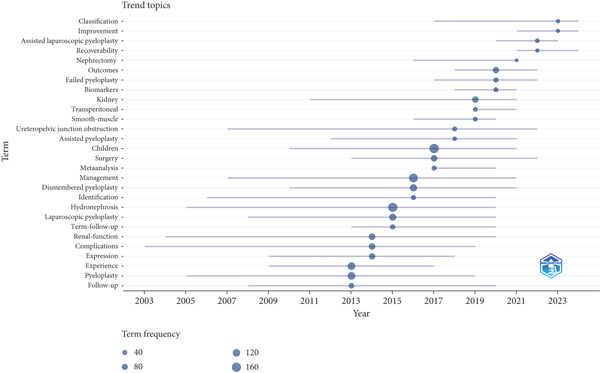
(b)

## 4. Discussion

Recent studies indicate that UPJO research has become a rapidly developing subspecialty in urology, particularly in pediatric urology [1, 2, 16]. Despite advances in UPJO treatment (e.g., multiple options and minimally invasive techniques), controversy remains over the optimal approach [16–18], and clinicians have long struggled with postoperative recurrence [19, 20]. Thus, a comprehensive analysis of UPJO literature is critical to clarify the global research status and identify future trends. This scientometric study identified and characterized all relevant publications on UPJO, and we sought to provide valuable information which may facilitate a better choice from diverse treatment options.

Several aspects of our analysis merit consideration. The published year ranged from 1948 to 2024, with the year 2009 manifesting as the most productive year. In the last seven decades, there has been a general growing trend in publications pertaining to UPJO. This growth was particularly prominent after 1990, driven by pivotal studies [15, 21]. Simultaneously, advances in diagnostic and monitoring imaging technologies, an area of intense research focus, were developed and disseminated [22, 23]. The most‐cited article in our analysis received 205 citations, fewer than the 454 citations of a landmark esophageal atresia study [13, 15]. This discrepancy may stem from differences in the size and focus of the respective professional communities. The *Journal of Urology*, *Journal of Endourology*, and *Urology* were the top three productive journals on UPJO, suggesting that the future of research in this area may be primarily focused on these journals. Although the top three journals do not have the highest IFs, they are among the few that focus exclusively on urology, thus contributing the most articles. Researchers specializing in UPJO may prioritize these journals.

Information on the influential publications serves as a beneficial index for scientometric study, a method that has been widely adopted in other research fields [24, 25]. The most cited article was released in 2006 by Decramer et al. in *Nature Medicine*, identifying polypeptides with the capacity to diagnose the severity of obstruction, subsequently validating said biomarkers in a prospective, blinded urine collection study [15]. This study contributes novel insights into the protein and peptide levels present within UPJO, which has the potential to facilitate a more nuanced categorization of disease subgroups and enhance the accuracy of clinical decision‐making processes concerning the selection of surgical candidates based on urinary protein biomarkers.

The United States was found to be the leading country in terms of total publications, citations, collaboration, and *h*‐index. In addition, authors and organizations from the United States exhibit a high degree of prevalence in key roles related to authorship and global collaboration networks. These results show that the United States has a strong scientific presence in the domain of UPJO studies, which has also been observed in other research areas, including robotic arthroplasty and congenital diaphragmatic hernia [7, 26]. The attainment of this accomplishment benefits from the optimal conditions conducive to basic science and clinical trials, encompassing advanced technology, professional researchers, and adequate financial resources. Moreover, previous studies have indicated that authors from the United States exhibited a preference for publishing and citing local articles [27, 28]. Despite the abundance of research on UPJO from China and India, the global collaboration among developing countries remains limited. The challenges of international cooperation, including temporal, financial, and integration issues, likely contribute to this limitation. On the other hand, this gap presents a critical opportunity to accelerate UPJO translational research. Translational progress relies on integrating diverse resources: High‐income regions offer advanced infrastructure, funding, and advanced tools, while lower income regions provide unique epidemiological data, understudied cohorts, and resource‐constrained management experiences [29]. Furthermore, recent studies have shed light on the complex dynamics of global research collaboration, especially in the context of low‐ and middle‐income countries (LMICs) [30, 31]. For instance, research has shown that while the overall number of international research collaborations has been increasing, the participation of LMICs remains disproportionately low in certain fields, including urology [32]. A recent study analyzed global research collaboration patterns across multiple disciplines and found that in natural science research, only 2.7% of articles published between 2015 and 2022 involved collaborations between higher income and lower income countries [33]. This lack of representation in research collaboration has far‐reaching implications for the field of UPJO research. LMICs often have unique patient populations, environmental factors, and healthcare infrastructure challenges that could offer novel insights into UPJO pathophysiology, diagnosis, and treatment. Fostering global collaborative efforts could offer numerous advantages, including improved patient recruitment, broader generalizability of results, scientific advancement, and increased citation impact [34, 35].

The results of keyword co‐occurrence analysis assist researchers in accurately catching research hot topics on UPJO. We found that “classification”, “improvement”, and “assisted laparoscopic pyeloplasty” may be new research hot topics in the field of UPJO. The management of UPJO poses a particularly challenging quandary for clinicians. While some children remain asymptomatic and exhibit spontaneous resolution of hydronephrosis over time, there are others for whom obstruction signifies considerable renal impairment. The challenge lies in distinguishing these groups and determining the optimal course of surgery [36]. Hence, recent studies have centered on the impact of the urinary tract dilation ultrasound classification system and the utilization of MRI, both in the prenatal and postnatal periods, for the stratification of risk in infants with prenatally diagnosed hydronephrosis. The objective of this stratification is to identify infants who are at risk of developing renal impairment or requiring surgical intervention [37]. The original OP reported by Anderson‐Hynes established the historical gold standard for the treatment of UPJO. The advent of laparoscopic techniques has led to the gradual predominance of LP as the prevailing standard in the field [38]. In comparison with traditional LP, RALP offers several notable advantages. Firstly, it provides a 3D stereoscopic field of view and a 10‐fold magnification of the conventional field, facilitating more precise positioning. Secondly, the vibration filtering feature of the robotic system contributes to enhanced stability during surgery. Thirdly, RALP boasts superior ergonomics, thereby reducing surgeon fatigue during procedures [39]. Finally, the learning curve of RALP is short, and it is significantly shorter in surgeons with considerable LP experience [40]. Clinically, this translates to tangible benefits: shorter learning curves for surgeons familiar with laparoscopic approaches, reduced operative fatigue during lengthy cases, and minimized trauma to surrounding tissues, all of which contribute to improved postoperative outcomes, including lower complication rates and faster recovery times in pediatric populations [41, 42]. Moreover, the rise of this keyword aligns with growing clinical demand for standardized, minimally invasive solutions for UPJO, particularly in managing complex cases where OP may carry higher morbidity. By identifying this trend, the present analysis underscores how technological innovation in surgical techniques is directly responding to the unique clinical needs of children with UPJO, bridging research output with frontline patient care. Therefore, the RALP is considered to be more appropriate for complex body cavity procedures, particularly reconstructive surgery. The limited spatial dimensions of the abdominal cavity in children necessitate more sophisticated procedures, and the dexterity of the robotic arm enables its operation in confined, narrow areas [43, 44]. We have reasons to believe that the increased promotion and application of RALP for the surgical management of UPJO will go forward in the foreseeable future.

This study is not without its limitations. First, it is possible that manuscripts published in other databases, such as Google Scholar or PubMed, were not included. Nevertheless, WoSCC, including approximately 34,000 core journals globally and spanning different disciplines, is distinguished as the most frequently utilized and appropriate database for scientometric analysis [45]. Second, the present study exclusively included original and review articles while excluding other types of papers, such as corrections or notes, despite their potential to offer valuable insights. Third, to focus specifically on UPJO‐related research, a “title”‐based search was employed rather than a broader “topic”‐based search. While this strategy may have excluded some pertinent publications, it is implausible that these omissions would substantially influence the overall analysis. Finally, the absence of a systematic collection of non‐English literature may potentially result in language bias, thereby overlooking the contributions of non‐English publications to the broader development.

## 5. Conclusions

This study identified all relevant UPJO publications and thoroughly characterized them. Our results show that interest in and understanding of UPJO have grown over seven decades, spanning multiple disciplines and incorporating innovative therapeutic strategies. Nevertheless, there is a paucity of global collaboration among developing countries. More international collaborations and translational research are required to further advance the area.

**Nomenclature**
IF: impact factor
LMICs: low‐ and middle‐income countries
LP: laparoscopic pyeloplasty
OP: open pyeloplasty
RALP: robot‐assisted laparoscopic pyeloplasty
UPJO: ureteropelvic junction obstruction
WoSCC: Web of Science Core Collection

## Conflicts of Interest

The authors declare no conflicts of interest.

## Funding

This study was funded by the Natural Science Foundation of Henan Province, 10.13039/501100006407, 252300421371.

## Data Availability

The data that supports the findings of this study is available from the corresponding author based on a reasonable request.
